# Biomechanical comparison of the non-locking bone plate, locking bone plate, and double-rod clamp internal fixation in a canine femoral model

**DOI:** 10.14202/vetworld.2025.773-781

**Published:** 2025-04-07

**Authors:** Rutjathorn Maneewan, Nattapon Chantarapanich, Takuma Morimoto, Chaiyakorn Thitiyanaporn

**Affiliations:** 1 Kasetsart University Veterinary Teaching Hospital, Faculty of Veterinary Medicine, Kasetsart University, Bangkok, 10900, Thailand; 2 Department of Mechanical Engineering, Faculty of Engineering Sriracha, Kasetsart University, Sriracha Campus, Laem Chabang, 20230, Thailand; 3 Department of Aerospace Engineering, Kochi University of Technology, Kami, 782-8502, Japan; 4 Department of Companion Animal Clinical Sciences, Faculty of Veterinary Medicine, Kasetsart University, Bangkok, 10900, Thailand

**Keywords:** bone plate, canine, clamp, femoral fracture, finite element analysis

## Abstract

**Background and Aim:**

Canine femoral fractures are prevalent in veterinary medicine, necessitating effective fixation methods to ensure stability and promote healing. Conventional bone plate fixation methods, including non-locking and locking plates, have inherent limitations, such as periosteal damage and mechanical failure. This study aims to evaluate the biomechanical performance of three fixation methods – non-locking bone plates, locking bone plates, and a novel double-rod clamp internal fixation system – using finite element analysis (FEA).

**Materials and Methods:**

A computed tomography-based canine femur model was created to simulate a midshaft commin-uted fracture with a 20 mm gap. Three fixation configurations were modeled: A non-locking bone plate, a locking bone plate, and a double-rod clamp system. FEA was performed to assess implant stress and proximal fragment displacement under physiological axial loading. Mesh refinement and multiple loading conditions were incorporated to enhance computational accuracy.

**Results:**

The non-locking bone plate exhibited the highest implant stress (1160.22 MPa), surpassing the material yield strength and indicating a risk of mechanical failure. The double-rod clamp system demonstrated lower stress (628.34 MPa), whereas the locking bone plate had the lowest stress (446.63 MPa). Proximal fragment displacement was highest in the non-locking bone plate (2.37 mm), followed by the double-rod clamp system (0.99 mm), with the locking bone plate exhibiting the least displacement (0.34 mm), suggesting superior stability.

**Conclusion:**

The double-rod clamp system emerged as a promising alternative, offering a balance between stability and stress distribution while minimizing periosteal damage. While the locking bone plate provided the greatest stability, the double-rod clamp fixation demonstrated favorable mechanical properties and could serve as a cost-effective and minimally invasive alternative in veterinary orthopedics.

## INTRODUCTION

Long bone fractures are common in small animal clinics. Traditional bone plate and screw fixation is widely used, but it is associated with complications such as periosteal damage and subsequent osteopenia, with reported incidence rates ranging from 7.1% to 20% [1]. Femur fractures account for 37% of long bone fractures in canines, making them one of the most frequently encountered orthopedic conditions [2]. Multiple forces from the muscle surrounding the femur bone can cause many fracture types, such as spiral, transverse, and comminuted fractures [3]. The goal of treatment is fixation using stable methods to allow animals to return to normal function. Bone plate and screw fixation is widely regarded as the gold standard for long bone fractures due to its superior stability and ability to promote effective bone healing. However, other techniques, such as external fixation and interlocking nails, are also used in specific clinical scenarios [4]. This technique involves securing a plate to the bone surface using screws, which facilitates alignment and stabilization of the fractured segments [5]. However, a major complication of bone plates and screws is the risk of periosteal damage. Because the plate must be in direct contact with the bone, blood flow to the periosteum is often restricted, leading to periosteal damage, compromised vascularity, and potential delays in bone healing [6]. At present, locking bone plates have been developed for fracture repair in canines, providing greater stability by eliminating the need for direct bone contact and thereby reducing periosteal compression and associated complications [7]. However, due to the high cost, the use of locking bone plates for bone surgery remains limited [8].

Despite advancements in fracture fixation techniques, existing devices still have notable limitations. These challenges highlight the need for a more cost-effective and minimally invasive alternative that reduces complications while maintaining sufficient stability. To address these issues, a novel internal fixation method using a double-rod clamp system was developed in this study. Unlike conventional fixation methods, the double-rod clamp system integrates a dual-rod structure that provides enhanced load distribution while minimizing periosteal compression. This study is the first to computationally validate the mechanical behavior of a plate compared with standard locking and non-locking plates using finite element analysis (FEA). The design consists of two longitudinal rods with clamps on each side of the segment. The longitudinal rods provide the strength and stability of the construct, whereas the clamps firmly secure the rods to the bone. This innovative design enhances fixation stability using fewer screws and allowing adjustable screw positioning, which reduces periosteal compression and minimizes tissue damage. The cost-effectiveness and adaptability of this device make it a promising solution for fracture treatment, providing a foundation for the future development of orthopedic fixation devices.

The Finite Element (FE) method is a numerical technique used to examine complex engineering and materials science problems by integrating mechanical, material science, and numerical methods [9]. The FE method simulates structures responding to various forces. This method produces detailed visualizations, often color-coded, to represent the stress distribution and compare the strength of different designs [10]. The key benefits of the FE method include the ability to predict material performance, optimize designs before creating prototypes, and assess product lifespan, leading to reduced experimental time and lower production costs [11].

Despite the widespread use of bone plates and screws as the gold standard for treating long bone fractures in veterinary orthopedics, these fixation methods are associated with notable limitations. Conventional non-locking bone plates often lead to excessive implant stress, increasing the risk of mechanical failure, while locking plates, though providing greater stability, remain costly and may contribute to stress shielding, leading to long-term bone weakening. Moreover, current fixation techniques frequently cause periosteal damage, compromising vascular integrity and delaying bone healing. Although alternative fixation systems, such as paraosseous clamp-cerclage stabilization and clamp-rod internal fixation (CRIF), have been introduced, they still present challenges, including suboptimal resistance to torsional loads and difficulty in achieving precise stabilization.

While biomechanical studies have extensively evaluated conventional plating systems, there is a lack of research on innovative, cost-effective, and minimally invasive alternatives that reduce periosteal compression while maintaining sufficient mechanical stability. The double-rod clamp internal fixation system presents a potential solution by incorporating a novel design that distributes stress more evenly and minimizes implant-related complications. However, its biomechanical performance has not been thoroughly validated through computational modeling or experimental studies. In addition, previous FEA studies on veterinary orthopedic implants have often overlooked the impact of multiple loading conditions, which are crucial for simulating realistic physiological stresses.

This study addresses these research gaps by employing FEA to comprehensively assess the mechanical behavior of the double-rod clamp fixation system in comparison to conventional non-locking and locking bone plates. By incorporating advanced computational modeling techniques, this research provides novel insights into the stress distribution, implant stability, and potential clinical applicability of this new fixation approach. Further experimental validation and clinical trials are necessary to determine its efficacy in real-world veterinary orthopedic applications.

The aim of this study is to evaluate the biomechanical performance of a novel double-rod clamp internal fixation system in comparison to conventional non-locking and locking bone plates in a canine femoral fracture model using FEA, with a focus on implant stress distribution, fracture stability, and potential clinical applicability.

## MATERIALS AND METHODS

### Ethical approval

This study used FEA to evaluate the mechanical performance of different bone fixation methods. Animals were not used in this study; therefore, ethical approval of animal use was unnecessary.

### Study period and location

This study was conducted from November 2022 to September 2024 at the Faculty of Veterinary Medicine, Kasetsart University, Bangkok, Thailand, and the Faculty of Engineering, Kasetsart University, Sriracha, Thailand.

### Model preparation

The femur bone model used in this study was derived from high-speed computed tomography (CT) (CT scanner; Optimal CT 660, GE Healthcare, USA) following standard segmentation protocols to ensure anatomical accuracy and to achieve the digital imaging and communications in medicine (DICOM) data at the Kasetsart University Veterinary Teaching Hospital, Bangkhen campus. The dog was diagnosed with other diseases that were unrelated to this study. The dog selected was 15 kg, mixed breed. The DICOM format files were processed using image processing software (3D slicer, www.slicer.org) [12] to create a stereolithographic (STL) file of the right femur. The cortical layer of the bone was obtained in the diaphysis region. In both epiphyses, the cortical layer was made to have a thickness of 3 mm. The cancellous bone was created inside the cortical layers. This STL file contains cortical and cancellous bone, which was examined in further studies. This STL file was used in further study. A 20-mm gap was introduced at the midshaft of the femur to simulate a comminuted fracture, based on clinical observations and prior biomechanical study by Field *et al*. [13], using Computer-Aided Design (CAD) software (VISI, Hexagon AB, Sweden).

### Fixation methods

**Figure 1:**
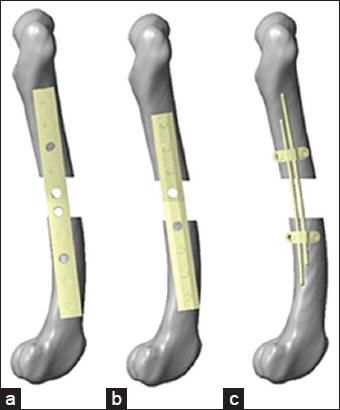
The three types of bone fixation devices: (a) Non-locking bone plate, (b) locking bone plate, (c) and internal clamp and rod fixation.

The experiment was divided into three groups using different bone fixation devices. The first group used a non-locking bone plate and six screws. The second group used a locking bone plate and six screws. The third group included the internal fixation of double-rod clamps, as shown in Figure 1. Three-dimensional models of the non-locking, locking bone plates, and screws of both systems were scanned using a 3D laser scanner (Artec Space Spider, Luxembourg). The scanned geometries were used as a reference to recreate the fixation modes in 3D CAD software (VISI, Hexagon AB).

The internal fixation of the double-rod clamp was designed using CAD software (VISI, Hexagon). This device includes a clamp that secures two metal rods, which are fixed to the bone with locking screws positioned above and below near the fracture site to increase its rigidity [14, 15].

**Figure 2:**
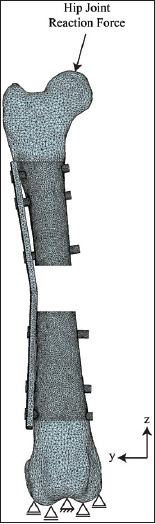
A meshed with the boundary condition of a non-locking bone plate with six screws assembled with a canine femoral gap model.

**Figure 3:**
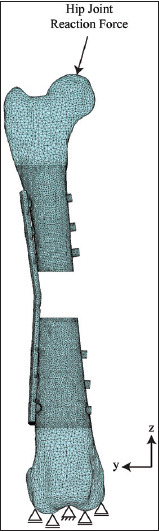
Meshed with the boundary condition of a locking bone plate with six screws assembled in a canine femoral gap model.

**Figure 4:**
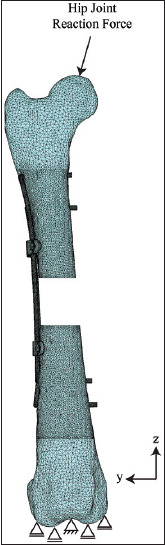
A meshed with the boundary condition of a double clamp and rod internal fixation assembled with a canine femoral gap model.

The FE models of the bone, rod clamp, plate, and screw were meshed with tetrahedral elements using FE preprocessing software (Patran, MSC Software, US). The numbers of elements and nodes of the FE models were as follows: non-locking plate, 369,219 and 88,653 (Figure 2); locking plate, 374,809 and 89,586 (Figure 3); and rod clamp, 518,171 and 124,090 (Figure 4). All material properties were assumed to be homogenous and isotropic. The material consecutive models were linearly elastic. The elastic modulus and Poisson’s ratio of each material is given in Table 1 [16].

**Table 1:** Material properties of the materials used in the FE model.

Material properties	Cortical bone	Cancellous bone	Stainless steel	Titanium
Young’s modulus (MPa)	15,000	350	193,000	110,000
Poisson’s ratio	0.3	0.25	0.3	0.35

FE=Finite element

A distinguishing feature of this FEA study was the incorporation of multiple loading conditions, including axial compression, bending, and rotational forces, to better simulate the realistic biomechanical environment of a canine femur. In addition, the mesh refinement strategy employed in this study improved the computational accuracy compared with previous veterinary orthopedics models.

All contacts between the fracture fragments and the implant were modeled as frictional contacts, except for the interface between the thread portion of the screw heads and the plate-locking screw holes, which were modeled as contacts [17]. This approach was adopted because the screw connections did not permit axial or shear movements. The friction coefficients for the implant-bone interfaces (0.3) and implant-implant interfaces (0.1) were selected based on previous FEA studies on orthopedic implants [18].

The testing was conducted using FEA by simulating realistic forces exerted on the femur bone by defining boundary conditions, including the magnitude and direction of the force applied to the femur bone model [19]. The fractured femur with the internal fixation system was loaded with a hip joint reaction force at the most superior surface of the femoral head with a magnitude of 238.78 N (X: −52.42 N Y: 10.1 N X: −232.74 N) [20]. The intercondylar notch surface was fully fixed to all degrees of freedom, whereas the femoral head was allowed only vertical translation due to hip joint socket constraint. Both condylar surfaces were used as bearing conditions. The FE simulation aimed to determine the displacement of the fracture site and the stress distributions of the femur, plate, rod, clamp, and screw [16].

## RESULTS

**Figure 5:**
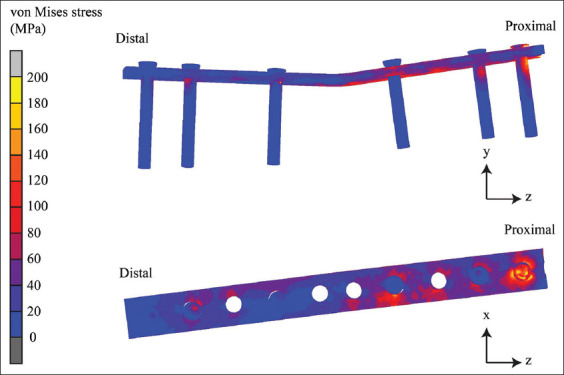
The von Mises stress contours of the non-locking bone plate.

**Figure 6:**
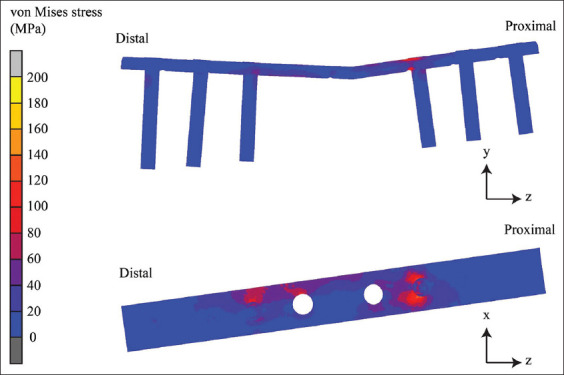
The von Mises stress contours of the locking bone plate.

**Figure 7:**
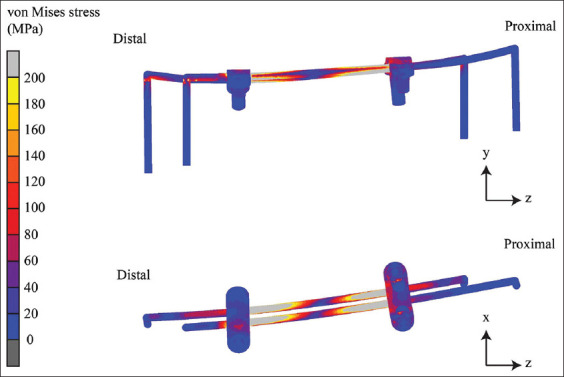
The von Mises stress contours of the internal fixation on the double-rod clamp.

The contours of equivalent von Mises stresses differ among implants and materials. This equivalent of a non-locking bone plate is shown in Figure 5, the locking bone plate in Figure 6, and the internal fixation of the double-rod clamp in Figure 7. It can be seen that the equivalent von Mises stress of the non-locking and locking plates is localized in the proximal segment, particularly the most proximal screw. The von Mises stress contours of the double-rod clamp internal fixation show high von Mises stress between working lengths.

The mechanical performances of the three bone fixation methods were analyzed in terms of implant stress and proximal fragment displacement under simulated load conditions. The maximum implant stress values were recorded for each fixation type, with significant differences observed between the non-locking bone plate and double-rod clamp internal fixation. The results show that the non-locking bone plate exhibited the highest implant stress of 1160.22 MPa, indicating a higher likelihood of implant failure under high-stress conditions. The double-rod clamp internal fixation exhibited a lower implant stress of 628.34 MPa, and in the locking bone plate, the implant stress was further reduced to 446.63 MPa, which is lower than the non-locking bone plate and the double-rod clamp internal fixation. In terms of displacement, the non-locking bone plate exhibited the highest displacement of 2.37 mm, exceeding clinically acceptable limits for stable fracture healing [10], the double-rod clamp internal fixation showed a displacement of 0.99 mm, indicating moderate stability, and the locking bone plate exhibited the lowest displacement of 0.34 mm, which remained more stable than the non-locking bone plate and the double-rod clamp system. The results are summarized in Table 2.

**Table 2:** FE results for each fixation type.

Fixation type	Material	Implant stress (MPa)	Proximal fragment displacement (mm)
Internal fixation of the double-rod clamp	Stainless steel	628.34	0.99
Non-locking bone plate	Stainless steel	1160.22	2.37
Locking bone plate	Titanium	446.63	0.34

FE=Finite element

## DISCUSSION

Bone plates and screws remain the gold standard for treating long bone fractures in veterinary practice due to their effectiveness in stabilizing fractures and promoting bone healing [21]. However, despite their widespread use, bone plates are associated with significant complications, including stress shielding, periosteal damage, and implant loosening [22]. Muroi *et al*. [22] have highlighted issues such as impaired periosteal blood flow and stress shielding, which lead to periosteal damage and delayed bone remodeling. Prolonged periosteal compression under conventional plates induces localized cortical bone thinning and increases osteoclastic activity, leading to implant-induced osteoporosis [6].

Locking plates were developed to address the limitations of conventional plates. Avoiding periosteal compression preserves blood flow and reduces the risk of implant-induced osteoporosis [13, 23]. The non-contact bone plate was first introduced in veterinary orthopedics in 1998. The locking head screw with monocortical fixation provides sufficient mechanical strength for long bone fracture fixation [24]. Research has compared the mechanical behaviors of a semi-contoured, locking compression plate-rod (LCP-rod) construct with a conventional anatomically contoured, limited-contact dynamic compression plate-rod construct. Results showed that, while both constructs displayed similar stiffness and stability under axial and cyclic loading, the LCP-rod construct offered improved angular stability [8]. This suggests that the use of locking plates could offer stability comparable to or even better than conventional plates. Similar to a previous study by Beirami *et al*. [25], the non-locking bone plate exhibited the highest von Mises stress, indicating an increased risk of implant failure, particularly under high-load conditions. However, while locking plates enhance angular stability, they do not fully eliminate complications like stress shielding, which occurs because the implant absorbs most of the load, reducing the natural mechanical stimulation necessary for bone remodeling. This phenomenon can result in bone weakening over time because the bone tissue does not receive the physiological stress necessary for maintaining strength and density [7]. In addition, a higher screw density near fracture sites enhances axial rigidity and elevates the stress concentration at the screw-plate interface, increasing the risk of implant failure [22].

The double-rod clamp internal fixation system is a novel approach for achieving rigid stabilization for treating long bone fractures. This innovative design combines the advantages of Paraosseous clamp- cerclage stabilization and CRIF. The system utilizes 316 L stainless steel rods available in different diameters that could be shaped in a U-shaped to anchor to the bone, coupled with special clamps to secure a U-shaped rod to the bone instead of wire binding, making it more effective than paraosseous clamp-cerclage stabilization. The clamp can be positioned anywhere along the rod to serve as a connector to prevent rod slippage. The posi-tion of the clamps can be adjusted as needed, depending on the fracture location, to ensure optimal stabilization at the fracture site. In this study, the positioning of the clamps in the double-rod clamp internal fixation system was carefully considered to optimize the stability.

Previous research by Chao *et al*. [26] has demonstrated that screw placement significantly affects fixation stability, with studies showing that screws positioned within 10 mm of the fracture site can optimize the load distribution and minimize stress concentration [26]. In addition, Vallefuoco *et al*. [27] have highlighted the importance of working length, which refers to the distance between the fracture site and the nearest fixation point. Distributing screws or clamps in a manner that maximizes construct stability and reduces stress concentration near the fracture [14]. Findings from biomechanical studies on bone plates for osteosynthesis indicate that the area nearest the fracture experiences the highest stress during load-bearing activities [28]. As a result, placing fixation points too far from the fracture may compromise the stability of the construction and increase the risk of failure due to stress accumulation [29]. To address this issue, our system positions the clamps approximately 10 mm from the fracture site. In addition, the distance between the clamp and the point at which the rod was bent to anchor to the bone was also maintained at 10 mm. This configuration ensures improved fracture stabilization while minimizing stress concentrations, providing optimal support to the fractured bone during the healing process. However, to enhance bone fixation stability, the number of clamps can be increased as needed.

This configuration offers superior strength due to the additional rods, enhancing overall rigidity and bending resistance while requiring fewer screws, which may reduce surgical time and decrease the risk of iatrogenic damage. This reduction in screw usage helps to minimize bone damage. Furthermore, the clamp design in the double-rod clamp internal fixation system allows screws to be inserted on both sides of the clamp, providing enhanced rod stabilization and reducing the risk of rod slippage. In contrast, the CRIF system’s clamps permit screw placement on only one side. Biomechanical comparisons between the limited contact dynamic compression plate with rod and CRIF systems indicate that CRIF exhibits reduced torsional stiffness under high torsional loads, suggesting that CRIF may be less resistant to torsional deformation, potentially limiting its use in cases where high torsional stability is essential [21]. The double-rod clamp internal fixation system aligns and stabilizes the bone along its longitudinal axis, acting as an extraosseous splint. The transcortical placement of the rods further enhances the resistance to rotational forces, offering a design advantage that may address the limitations of the torsional stiffness observed in CRIF systems [21, 30].

This device is a cost-effective alternative and reduces the complications commonly associated with paraosseous clamp-cerclage stabilization, CRIF, and the bone plate. The double-rod clamp system allows minimal movement at the fracture line due to the rods’ flexibility. This controlled micro-movement promotes callus formation, a critical process in natural bone healing. In addition, the system’s small implant dimensions minimize disruption to bone tissue, preserve blood circulation, and protect surrounding soft tissues. These features make it a minimally invasive and biologically friendly alternative for fracture treatment [31, 32]. One critical advantage of the double-rod clamp system is its adaptability to different bone geometries and fracture types. Unlike locking plates that require precise anatomical contouring, this system allows more flexibility in fracture fixation. In addition, the reduced number of screws may decrease surgical time and limit the risks associated with excessive bone drilling. These features make the system particularly suitable for cases in which cost-effectiveness and minimally invasive approaches are priorities in veterinary practice.

According to our study, double-rod clamp internal fixation demonstrated moderate stress and displacement, with stress values and proximal displacement lower than those observed in non-locking bone plates. This is likely due to the design featuring two U-shaped rods that anchor securely to the bone, increasing the contact interface between the rods and the bone. The enhanced interface improves the load distribution and reduces the risk of implant failure during the healing process [33]. Overall, the double-rod clamp system is a promising alternative to traditional bone plates.

Although developed for veterinary applications, the principles underlying the double-rod clamp system align with certain human orthopedic techniques, such as hybrid external fixation. The insights gained from this study could inform future research on minimally invasive fracture stabilization in human medicine, particularly in low-resource settings where cost-effective alternatives to locking plates are needed.

The use of the double-rod clamp system has some limitations. The strength of this system depends on the pin size; if pins larger than 2 mm are used, they become difficult to bend into a U-shape, resulting in reduced strength of the fixation [34]. Our study found that the stress experienced by the double-rod clamp internal fixation was greater than the stress on the locking bone plate. It also exhibited lower stability, as evidenced by greater proximal displacement. Therefore, a locking bone plate may be more appropriate in cases requiring high stability or in older patients. However, the use of the double-rod clamp system remains another option for cases involving younger patients and is suitable for fractures requiring micromovement to promote callus formation. In addition, it is suitable for cases in which cost efficiency is a priority.

This study compared the mechanical efficiency of the double-rod clamp internal fixation method with the use of locking and non-locking bone plates by FEA to provide insights into the biomechanical performance under various load conditions. The results show that using a locking bone plate provides superior stability and reduces stress at the fracture site, followed by double-rod clamp internal fixation in terms of stability, with the non-locking bone plate showing the least stability among the three fixation methods.

The results of this study demonstrate the mechanical performance of a double-rod clamp internal fixation system using FEA. In addition, biomechanical testing using a universal mechanical testing machine and clinical studies should be conducted to confirm the results of this study. Further study could also be extended to other long bones, such as the tibia and humerus, to explore the broader potential of this system in orthopedic applications.

## CONCLUSION

This study evaluated the mechanical performance of the double-rod clamp internal fixation system in comparison to locking and non-locking bone plates using FEA. The results indicated that the double-rod clamp system provides moderate stability and experiences lower implant stress than non-locking plates, suggesting its potential as an alternative fixation method for fractures requiring controlled micromotion to facilitate callus formation. This characteristic makes it particularly suitable for younger animals or cost-sensitive treatment scenarios. Although locking plates demonstrated superior stability, the double-rod clamp system offers notable advantages, including greater adaptability, reduced periosteal damage, and a lower risk of stress shielding.

To confirm its clinical viability, further mechanical testing and *in vivo* trials are necessary. In addition, advancements in material selection and implant design could further enhance the effectiveness of the double-rod clamp system. The use of titanium alloys or bioresorbable materials may improve long-term biocompatibility, while patient-specific 3D-printed implants could provide customized fixation solutions tailored to individual anatomical variations. These innovations may extend the clinical applications of the double-rod clamp system beyond veterinary orthopedics, offering broader implications for fracture management in both animal and human medicine.

## AUTHORS’ CONTRIBUTIONS

NT and CT: Designed the new fixation device model. NT: Designed the experiments. RM and TM: Contributed to the refinement of the structure of the bone fixation device. RM: Conducted the experiments and drafted the manuscript CT: Edited the manuscript. All authors have read and approved the final manuscript.
